# On Navigating Analytical Choices in Research on Early Life Adversity: A Commentary on Sisitsky et al. (2023)

**DOI:** 10.1007/s10802-023-01103-7

**Published:** 2023-08-03

**Authors:** Charlotte A. M. Cecil, Isabel K. Schuurmans

**Affiliations:** 1 Department of Child and Adolescent Psychiatry and Psychology, Erasmus MC University Medical Center Rotterdam, PO Box 2040, 3000 CA Rotterdam, the Netherlands; 2 Department of Epidemiology, Erasmus MC University Medical Center Rotterdam, Rotterdam, the Netherlands; 3 Molecular Epidemiology, Department of Biomedical Data Sciences, Leiden University Medical Center, Leiden, the Netherlands; 4 The Generation R Study Group, Erasmus MC University Medical Center Rotterdam, Rotterdam, the Netherlands

**Keywords:** Early life adversity, Threat, Deprivation, Child mental health, Stress

## Abstract

Early life adversities (ELA), including exposure to childhood maltreatment, deprivation or community violence, rarely occur in isolation. This co-occurrence poses several conceptual and methodological challenges for researchers, who must decide how best to model ELA and its association with outcomes. In this commentary, we discuss how different analytical choices come with their own – often complementary – sets of assumptions, strengths and limitations, which should be carefully considered when designing research on ELA. We then summarize work published in this issue by Sisitsky et al. (Research on Child and Adolescent Psychopathology, 2023), which serves as an important example of how different approaches can be incorporated in research in order to capture ELA as a complex phenomenon, while generating actionable results. Ultimately, such integration can enhance the quality and relevance of research, contributing to a more comprehensive understanding of ELA and its effects on health outcomes, paving the way for more targeted prevention and intervention strategies to promote children’s wellbeing.

## Introduction

Exposure to early life adversity (ELA) is among the strongest predictors of poor child and adolescent mental health, as well as increasing risk for negative cognitive, social and physical health outcomes later in life. Despite this well-established link, mapping how specific early life adversities (e.g., child abuse and neglect, socio-economic deprivation and community violence) relate to adverse health outcomes has been far from straightforward, as both equifinality (i.e., multiple adversities associating with the same outcome) and multifinality (the same adversity associating with multiple outcomes) represent the norm rather than the exception**.** This tangled web of ELA-outcome associations currently complicates efforts to design effective prevention and early intervention strategies to curb the negative impact of ELA.

A major challenge in disentangling this web of associations is the tendency of adversities to *co-occur* with one another; in other words, individuals exposed to one type of early life adversity often report experiencing additional adversities (Dong et al., 2004). This co-occurrence is evidenced by the known correlations between different types of child abuse and neglect, as well as by correlations between exposure to domestic and community violence. As a result, researchers are confronted with the question of how to best model ELA and its relationship with (mental health) outcomes. Two main considerations include: (1) whether to focus on single adversities or broader multi-adversity models; and (2) whether to examine associations between ELA and outcomes using variable-centered or person-centered approaches. In this commentary, we discuss each of these methodological considerations and advocate for their integration within ELA research, drawing insights from the work of Sisitsky et al. (2023) published in this issue.

## How to Measure ELA: Differentiating Between Specific, Cumulative and Dimensional Models

Historically, studies on ELA have primarily focused on individual adversities (e.g., physical abuse *or* sexual abuse; childhood maltreatment *or* community violence) – and their effects on health outcomes – in isolation. This approach, known as the **specificity model**, offers the advantage of a clear focus, which facilitates translation into public health recommendations, by pinpointing specific targets for prevention and early intervention. Although narrow in breadth, the specificity model also makes it more feasible to delve ‘deeper’ into each exposure, for example by using more detailed assessments, adopting a multi-rater approach, or by examining the role of exposure characteristics such as timing, severity and chronicity. However, investigating single adversities without considering their co-occurrence with other exposures lacks ecological validity and comes with significant drawbacks. Perhaps most consequential is the risk for biased and overinflated effect estimates, as observed associations may at least in part be explained, compounded or modified by other (unmeasured) adversities. Additionally, the specificity model does not account for the fact that individuals exposed to the same adversity (e.g., physical abuse) likely vary in their exposure to other adversities (e.g., physical neglect), which could contribute to outcome heterogeneity – an important factor to consider both from an etiological standpoint as well as for the development of personalized intervention strategies.

On the opposite end of the ELA ‘measuring spectrum’ lies the **cumulative model**, which has gained increasing popularity since the seminal work on adverse childhood experiences (ACEs) by Felitti et al. (1998). This approach involves summing different adversities into a single cumulative (i.e., total) ELA score, allowing researchers to obtain a broad overview of the overall burden of adversity experienced by individuals, while still adopting a straightforward strategy that simplifies complex data (Evans et al., 2013). This cumulative model has proven valuable in showing that ELA associates with outcomes following a ‘dose-response’ relationship (i.e., the higher the number of adversities experienced, the worse the outcome), a finding replicated by numerous studies. However, the practicality of the cumulative model comes at the cost of precision: the model implicitly assumes a univariate structure, whereby all adversities contribute equally to the score, leading to an inevitable loss of specificity regarding the unique contributions of individual adversities (McLaughlin & Sheridan, 2016). Individuals with the same overall ELA score may present with very different exposure combinations and outcome profiles, complicating efforts to map ELA-outcome associations. Furthermore, combining a wide range of different adversities together obscures any temporal or causal relationships between them, as well as the potentially distinct mechanisms through which they may influence outcomes.

To address these limitations, a **dimensional model** has been proposed, which recognizes that adversities may cluster along different dimensions based on shared features. Of these, one of the most prominent is the ‘threat-deprivation’ model (McLaughlin & Sheridan, 2016), which groups adversities into two separate dimensions: exposure to *threat* (i.e., harm or the threat of harm; for example physical abuse and community violence) versus *deprivation* (i.e., an absence of expected environmental input; for example physical neglect and lack of stimulation). Support for this model includes evidence that neurobiological outcomes can differ for individuals exposed to threat versus deprivation, suggesting at least partially distinct developmental adaptations (e.g., poor discrimination of threat and safety cues during fear-conditioning for those highly exposed to threat; disruptions in the neural circuitry that supports reward learning for those highly exposed to deprivation; (McLaughlin et al., 2014)). The dimensional model lies at the intersection of the specificity and cumulative models in terms of its advantages and disadvantages: it accounts for co-occurring adversities and decreases multiple-testing burden by reducing ELAs to a smaller number of dimensions, while maintaining some degree of specificity by distinguishing between ELA dimensions based on common features and potentially shared underlying mechanisms. However, the model may still oversimplify ELA experiences, leading to arbitrary decisions on how to classify exposures. This can be particularly challenging for ELAs that can present a blend of both dimensions, such as parental psychopathology or substance abuse.

## How to Model ELA-outcome Associations: Using Variable Versus Person-centered Approaches

Having navigated analytical choices in the measurement of ELA, researchers are typically confronted with another set of decisions on how to model *associations* between ELA and health outcomes. Traditionally, the most common strategy has been to employ **variable-centered approaches**, where the focus is on how individual variables relate to each other within an entire sample (e.g., the relation between early life adversity and depressive symptoms). Variable-centered approaches are relatively straightforward to implement across different populations, facilitating replication and meta-analytic efforts. These approaches also offer flexibility in statistically testing for potential confounders, mediators and moderators of ELA-outcome associations. In terms of translational potential, variable-centered approaches enable the identification of specific risk or protective factors robustly associated with health outcomes. This, in turn, can provide helpful insights for public health policies and prevention programs, and can contribute to the development of screening tools which use ELA variables to identify those at risk for negative outcomes at a *population level*. However, in doing so, variable-centered approaches assume sample homogeneity; in other words, that the relationship between variables is constant across all individuals in a given population. This assumption can be problematic as it overlooks potential heterogeneity in patterns of co-occurring ELAs within the population, resulting in a loss of information at the individual-level (Jobe‐Shields et al., 2015).

In contrast,** person-centered approaches** place their emphasis on identifying meaningful subgroups of individuals *within* a population. These data-driven methods (e.g., latent profile or latent class analyses) allow researchers to identify distinct subgroups of individuals who cluster together according to key variables of interest – such as their patterns of adversity exposure (Spurk et al., 2020). This can lead to a more nuanced understanding of individual differences within a population, as well as insights into how different adversities may interact to produce certain outcomes (Jobe‐Shields et al., 2015). As a result, person-centered approaches are well suited for the identification of high-risk groups that may benefit from targeted interventions, therefore promoting precision medicine and individualized care. However, it can be difficult to distinguish true versus spurious subgroups, for example when the model is misspecified, when data is non-normally distributed, or when indicators are non-linearly related (Spurk et al., 2020). Even if identified subgroups adequately characterize the population from which they are estimated, they may lack generalizability, which can make it difficult to apply findings from a person-centered analysis to a different population. Moreover, in the event that identified subgroups reflect true and generalizable subgroups, important relationships between variables can still be missed, as person-centered approaches do not necessarily reveal the nature of the relationship between variables that cluster within a subgroup.

## An Integrated Approach to Investigate Associations Between Co-Occurring Early Life Adversities and Child Mental Health Outcomes: Contributions of Sisitsky et al. (2023) to the Field

An application of these analytical choices can be found in this issue, as performed by Sisitsky et al. (2023). The authors utilized data from a population-based birth cohort of mostly racial and ethnic minority youth, generally exposed to heightened levels of ELA, who were born between 1998 and 2000 across 20 large cities in the United States (the Future of Families and Child Wellbeing Study, N = 2,483, 51.6% male). ELA was measured using a range of variables when children were 3 years old, including parent-rated reports as well as community-level statistics, and subsequently combined into a broader multi-adversity model. Interestingly, confirmatory factor analyses showed that a unidimensional model (similar to a **cumulative** model) fit the data poorly, while a two-dimensional model of ELA (similar to the threat-deprivation **dimensional** model) showed a marginal fit. The optimal solution consisted of a four-dimensional model, differentiating between home threat, community threat, neglect, and lack of stimulation. This indicates that, in some cases, an approach that retains more specificity in adversities can better capture variance within ELA, as compared to higher-order solutions. However, a key difference with the traditional **specificity** model – which typically focuses on one single adversity – is that here multiple adversities were still examined in a comprehensive manner, allowing to model the co-occurrence of ELAs. As a next step, a **person-centered** approach was used to examine associations between the four ELA-dimensions and child biopsychosocial outcomes at age 9. These analyses identified 8 distinct subgroups based on unique patterns of exposure to home threat, community threat, neglect, and lack of stimulation. While 5 of the subgroups were characterized by the levels of a single ELA dimension (e.g., community threat), the other 3 subgroups (collectively representing over half of the sample) showed varying levels across multiple ELA dimensions. This suggests that the specificity model may work best for some individuals, but misses the more complex patterns of ELA experienced by others. In turn, these subgroups were found to be differentially associated with internalizing and externalizing behaviors (but not telomere length), indicating that, as the authors put it, “*it is not just the amount of ELA, but the combination of exposures that predict child mental health outcomes*”. Notably, the authors also ran associations using **variable-centered** analyses for comparative purposes. These generally produced consistent results in terms of identifying unique associations between specific adversities and outcomes, however were less informative regarding the impact of heightened exposure to multiple adversities.

Overall, the study of Sisitsky et al. (2023) provides an important example of how different analytical approaches can be integrated to better understand the complexity of ELA and its associations with child mental health, while still producing findings that have the potential to inform public health policies and intervention strategies. A key message that emerges from this work is that one size *does not* fit all – in many cases, population-level screening tools that focus on exposure to single adversities may suffice to identify at-risk individuals; however, these strategies should be complemented with a more personalized approach to capture those with complex ELA profiles, in order to improve risk prediction and offer more tailored support. Nonetheless, caution should be exercised when interpreting the identified subgroups and their outcomes, as the subgroups were derived from a specific cohort and may not generalize to other populations, and as such await replication. Further, exposures and outcomes were measured at a single time point, which may obscure important developmental dynamics in the relationship between ELA and mental health. Indeed, timing and chronicity of exposure to adversity are important, but understudied factors in ELA-outcome associations, due to challenges in measuring these characteristics reliably and the limited availability of cohorts with repeated ELA data. In this context, it is noteworthy that childhood adversities are often preceded by prenatal adversities (e.g., maternal exposure to stressful life events or psychopathology), as evidenced by the known stability of risk factors across these developmental periods. This means that observed effects of childhood adversity on outcomes may be partly due to exposures occurring before birth, and conversely, that prenatal effects on outcomes may be partly mediated by adversities during childhood. Despite this, information on prenatal and postnatal ELA are rarely studied simultaneously, pointing to an important avenue for future research.

## Conclusions

In conclusion, researcher face many analytical considerations when studying the tangled web of associations between co-occurring early life adversities and child (mental health) outcomes. The models and approaches discussed in this commentary all have idiosyncratic strengths and limitations; the choice between them therefore depends on the research questions, available data and sample size, and the level of detail required to address the research objectives effectively. In essence, a trade-off exists between approaches that prioritize practicality and parsimony versus those that aim to comprehensively model complex constructs, as well as those that focus on the population versus the individual. Integrating these complementary approaches can help to strike a balance between trade-offs in order to reach a more nuanced and complete understanding of ELA while still delivering actionable results to guide public health policies and interventions to improve child well-being.
